# Exercise duration modulates upper and lower respiratory fluid cellularity, antiviral activity, and lung gene expression

**DOI:** 10.14814/phy2.15075

**Published:** 2021-10-21

**Authors:** Safwan K. Elkhatib, Jessica Alley, Michael Jepsen, Laurel Smeins, Andrew Barnes, Shibani Naik, Mark R. Ackermann, David Verhoeven, Marian L. Kohut

**Affiliations:** ^1^ Department of Kinesiology Iowa State University Ames Iowa USA; ^2^ Program of Immunobiology Iowa State University Ames Iowa USA; ^3^ Department of Veterinary Pathology College of Veterinary Medicine Iowa State University Ames Iowa USA; ^4^ Department of Veterinary Microbiology and Preventive Medicine College of Veterinary Medicine Iowa State University Ames Iowa USA; ^5^ Present address: Cellular & Integrative Physiology College of Medicine University of Nebraska Medical Center Omaha Nebraska USA; ^6^ Present address: Lineberger Comprehensive Cancer Center, School of Medicine University of North Carolina Chapel Hill North Carolina USA; ^7^ Present address: College of Osteopathic Medicine Campbell University Lillington North Carolina USA; ^8^ Present address: Kirksville College of Osteopathic Medicine A.T. Still University Kirksville Missouri USA; ^9^ Present address: Arisan Therapeutics 11189 Sorrento Valley Rd, Suite 104, San Diego California USA; ^10^ Present address: Director, Anatomic Veterinary Pathology Diagnostics Zoetis Clear Lake Iowa 50428 USA

**Keywords:** exercise, fatigue, influenza A, lung gene expression, mucosal immunity, nasal lavage fluid

## Abstract

Exercise has substantial health benefits, but the effects of exercise on immune status and susceptibility to respiratory infections are less clear. Furthermore, there is limited research examining the effects of prolonged exercise on local respiratory immunity and antiviral activity. To assess the upper respiratory tract in response to exercise, we collected nasal lavage fluid (NALF) from human subjects (1) at rest, (2) after 45 min of moderate‐intensity exercise, and (3) after 180 min of moderate‐intensity exercise. To assess immune responses of the lower respiratory tract, we utilized a murine model to examine the effect of exercise duration on bronchoalveolar lavage (BAL) fluid immune cell content and lung gene expression. NALF cell counts did not change after 45 min of exercise, whereas 180 min significantly increased total cells and leukocytes in NALF. Importantly, fold change in NALF leukocytes correlated with the post‐exercise fatigue rating in the 180‐min exercise condition. The acellular portion of NALF contained strong antiviral activity against Influenza A in both resting and exercise paradigms. In mice undergoing moderate‐intensity exercise, BAL total cells and neutrophils decreased in response to 45 or 90 min of exercise. In lung lobes, increased expression of heat shock proteins suggested that cellular stress occurred in response to exercise. However, a broad upregulation of inflammatory genes was not observed, even at 180 min of exercise. This work demonstrates that exercise duration differentially alters the cellularity of respiratory tract fluids, antiviral activity, and gene expression. These changes in local mucosal immunity may influence resistance to respiratory viruses, including influenza or possibly other pathogens in which nasal mucosa plays a protective role, such as rhinovirus or SARS‐CoV‐2.

## Graphical abstract

Exercise duration differentially alters the cellularity of respiratory tract fluids, antiviral activity, and lung gene expression. The change in nasal fluid leukocytes correlated with the post‐exercise fatigue rating after 180 min of exercise. These changes in local mucosal immunity may influence resistance to respiratory viruses, including influenza or possibly other pathogens in which nasal mucosa plays a protective role, such as rhinovirus or SARS‐CoV‐2.

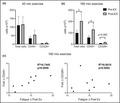

## 1 INTRODUCTION

The health benefits of moderate regular exercise are widely recognized. However, the results of prior studies suggest that a single session of prolonged, intense exercise might increase susceptibility to respiratory infection. For example, several epidemiological studies reported an increased incidence of infection following prolonged endurance events, such as running a marathon or ultramarathon (Nieman et al., 1990; Peters, 1997; Peters & Bateman, 1983; Peters et al., 1993). The findings from one study suggested that individuals who experienced an infection before an endurance event were at increased risk for infection post‐event (Ekblom et al., 2006), raising the possibility that recent illness may increase the risk of infection. The concept that an "open window" of vulnerability to infection may occur following heavy intense exercise was proposed quite some time ago, and many studies have documented changes of immune response after this type of exercise, lasting for hours to several days (Kakanis et al., 2010). More recent metabolomic and proteomic analyses also demonstrated significant changes across various immune parameters following exercise [reviewed thoroughly elsewhere (Nieman et al., 2019)].

However, the concept of a post‐exercise "open window" leading to increased infections has been called into question (Campbell & Turner, 2018). Also, there is substantial debate as to whether upper respiratory symptoms reported by athletes are caused by infectious pathogens, or instead are allergic responses or other effects on the airways (Campbell & Turner, 2018; Walsh & Oliver, 2016). And rather than the physical exertion of an endurance event, other factors often accompany competitions: travel, exposure to crowds and pathogens, psychological stress, lack of sleep, or suboptimal nutrition, and may influence the risk of infection. These factors are challenging to tease apart in studies with human subjects. In contrast, it is possible to control additional stressors and exposure to pathogens in animal models. In studies comparing moderate and prolonged exercise immediately prior to pathogen exposure, prolonged strenuous exercise (2.5–3.5 h) resulted in increased morbidity and mortality from a viral challenge (Davis et al., 1997) that could be due in part to reduced interferon‐β production (Kohut et al., 1998). Exercise to volitional fatigue just prior to influenza exposure also increased morbidity and mortality (Murphy et al., 2008), as did prolonged, strenuous exercise after infection with influenza virus (Ilbäck et al., 1984; Martin et al., 2009). In contrast, moderate exercise performed before viral challenge or early in the course of viral infection may in some instances reduce morbidity or viral load (Davis et al., 1997; Martin et al., 2009; Sim et al., 2009). Therefore, when variables are better controlled, as in animal studies, the data generally suggest that intense, fatiguing exercise can increase the risk of severe infection, providing some support for the open window theory.

Further investigations into potential mechanisms underlying susceptibility to infection in the context of exercise could significantly advance our understanding of the risks or benefits associated with endurance exercise. Most studies involving human participants rely solely on blood‐based measures to assess immune responses, while the types of infections commonly associated with exercise are respiratory tract infections. Therefore, it is imperative to examine host defenses at the site of potential infection. Measures of mucosal immunity in humans have largely focused on salivary immunoglobulin A (IgA), and studies generally suggest reduced salivary IgA is correlated with a greater risk of infection (Gleeson et al., 1999), although this is not a consistent finding. Saliva has also been used to assess the effect of exercise on antimicrobial proteins or peptides (AMPs) (Allgrove et al., 2014; Ligtenberg et al., 2015; West et al., 2010). The nasal mucosa is less well‐studied in the context of exercise, but essential host defense activity against respiratory pathogens occurs in the nasal mucosa of the upper airways. In addition to leukocytes that contribute to the host defense of the upper airways, epithelial cells exhibit various antimicrobial and immunomodulatory functions (Iwasaki et al., 2017; Vareille et al., 2011). The nasal epithelial cells play an important role in infection and host defense for a range of viral pathogens including influenza, SARS‐CoV‐2, and rhinovirus (Chen et al., 2019; Gallo et al., 2021; Mihaylova et al., 2018; Yan et al., 2016). Therefore, a better understanding of the impact of exercise duration on nasal epithelial cells and recruited immune cells may be important in evaluating host protection from infection. Nasal mucus also contains many AMPs with antiviral or antibacterial properties, and recent studies of the nasal mucus proteome identified a breadth of proteins, many of which have yet unknown effects on immunity (Tomazic et al., 2020). Importantly, upper airways may serve as a reservoir for microbial colonization and subsequent descending infection to lower airways. Overall, there is extremely limited information concerning nasal mucosal immunity and exercise, whereas the effect of exercise on lower airways has received greater attention due to the focus on exercise‐induced bronchoconstriction (EIB).

In this investigation, we sought to evaluate the effect of exercise duration on nasal mucosal host defense in human participants and examine how exercise duration may influence host defense in the lung with a mouse exercise model. Based on the open window theory of increased vulnerability to respiratory infection following prolonged exercise, we hypothesized that 180 min of exercise would be associated with reduced nasal lavage fluid (NALF) antiviral activity relative to no exercise or 45 min of exercise. Furthermore, based on findings suggestive of bronchial epithelial cell damage and increases in inflammatory cells following long‐term endurance exercise (Morici et al., 2016), we hypothesized that 180 min of exercise would be associated with epithelial cell loss and increased cellular stress and inflammatory gene expression in the lower airways. Given the invasive procedures involved in evaluating human lower respiratory tract function, a mouse model of exercise was used in these studies. Our previous work demonstrated increased morbidity and mortality from respiratory viral infection following 180 min of exercise and suggested that 45 min of exercise conferred protection (Davis et al., 1997). Therefore, we evaluated the effect of 45, 90, or 180 min of exercise on immune cell populations and gene expression in the lower respiratory tract. The primary genes of interest included cellular stress, inflammation, and apoptosis‐related genes. Heat shock proteins (HSPs) are a class of cellular stress proteins that may be induced by exercise, with the levels of expression related to exercise intensity and duration (Henstridge et al., 2016). HSPs also have a role in immune regulation and may link cellular stress or host defense signaling with immune response (Krüger et al., 2019; Muralidharan & Mandrekar, 2013), although much less is known with respect to exercise and HSPs in the respiratory tract. The cross talk between HSPs and immune response (Calderwood et al., 2016) might play a role in respiratory host protection following exercise. Given the evidence of respiratory epithelial cell damage that may accompany exercise (Chimenti et al., 2007, 2010; Combes et al., 2019), we used a pathway‐targeted gene microarray that included measures of cellular stress, inflammation, and apoptosis‐related genes.

## 2 METHODS

### 2.1 Participants

Healthy males (*n* = 10) and females (*n* = 8) between the ages of 18 and 60 who were free of chronic disease were eligible for participation in the study. Eligibility criteria included regular participation in moderate‐intensity exercise at least 4 days a week, with two or more sessions consisting of ≥45 min in duration. Moderate‐intensity exercise was defined as an intensity corresponding to at least 60% of maximal heart rate or equivalent rating on the Borg Rating of Perceived Exertion (RPE) scale of 12–14. Individuals with any condition that could impact mucosal immunity or who were currently taking a medication that could influence mucosal immunity, such as a nasal corticosteroid spray, were not eligible. The use of tobacco products was not an exclusion criterion, however, none of the participants reported using tobacco on the initial health screening questionnaire. Two exercise conditions (45 or 180 min of exercise) were included in the study. Although inclusion/exclusion criteria for participation in either exercise condition were identical, not all subjects elected to participate in both exercise conditions. The study was approved by the Institutional Review Board (IRB) and carried out in accordance with all IRB guidelines.

### 2.2 Nasal lavage, exercise, and fatigue measure

Nasal wash fluid was collected under three conditions during separate visits: at rest, after 45 min of moderate‐intensity cycling or running exercise, or after 180 min of moderate‐intensity cycling or running exercise. Visits were separated by at least 4 days and up to 1 week apart. Prior to each visit, participants completed a brief survey to identify any symptoms of allergy or illness present during or in the week prior to the visit. If symptoms were present, participants were asked to postpone the visit until 1 week after symptom resolution. For nasal lavage, 10 ml of sterile saline was instilled into each nostril using a syringe fitted to the nasal opening to prevent backflow. This fluid then drained through the contralateral nostril and was collected in a sterile petri dish placed directly below the nostril. Participants were instructed to avoid swallowing the saline, and gently blow any remaining saline into the petri dish by occluding the nostril in which the saline was instilled. Contents of the petri dish were transferred to a centrifuge tube after filtration through a 100 µm cell strainer, followed by centrifugation at 300 *g* at 4°C. Supernatant was removed and stored at −80°C until subsequent analysis in antiviral assays. Cell pellets were resuspended in FBS stain buffer (BD Biosciences) and stored on ice during processing. After the saline rinse, a FLOQSwab (Copan Diagnostics Inc.) was inserted into the nostril, followed by a gentle brush of nasal lining. Swabs were placed in centrifuge tubes containing sterile, supplemented RPMI medium (1% penicillin and streptomycin), and gently swirled to release cells. Samples were centrifuged for 10 min at 300 *g* at 4°C, followed by removal of the supernatant, with remaining cells then resuspended in FBS stain buffer until preparation for flow cytometry.

In the rest condition, participants rested quietly for 20–30 min prior to nasal lavage. In the exercise conditions (45 or 180 min), the Karvonen formula [[((220 − age − resting heart rate) × 0.55) + resting heart rate] for heart rate low end of range, and [((220 − age − resting heart rate) × 0.65) + resting heart rate] for heart rate high end of range] was used to estimate target heart rate within the moderate‐intensity range (55%–65%). Participants were permitted to select either cycle ergometer or treadmill exercise consistent with which the exercise type they were most familiar. Resting heart rate and blood pressure were measured after 15 min of quiet rest prior to exercise onset. The first 5 min of each exercise session involved a gradual increase in exercise intensity until desired heart rate intensity was achieved. Heart rate, blood pressure, and Borg scale RPE were measured every 15 min. In the 45‐min exercise condition, fatigue was measured before exercise onset and during the final minute of exercise using a visual analog fatigue scale of 1 (no fatigue) to 10 (maximal fatigue). During the 180‐min exercise session, heart rate, blood pressure, and relative perceived exertion were assessed every 15 min, and the fatigue scale was completed every 30 min. The fatigue scale consisted of a 24 cm line with the number 1 at the left end of the line and the number 10 at the right end of the line. Subjects were directed to place a mark on the line indicating the current level of fatigue, with 1 representing no fatigue and 10 representing maximal fatigue. The difference in distance from pre‐exercise to post‐exercise was determined. During either exercise session, workload was adjusted periodically to maintain heart rate within the target range.

### 2.3 Leukocyte and epithelial cell assessment

Cells obtained as described above from the nasal lavage fluid and the nasal swab were combined and subsequently counted. Cells were incubated with Human BD Fc block (BD Biosciences) for 10 min, followed by 0.045 µg PE‐Cy5 anti‐human CD45 (Leukocyte common antigen; clone HI30, Biolegend) and 0.1875 µg PE anti‐human CD326 (epithelial cell surface antigen; clone C4, Biolegend) in a 100 µl volume. Cells were incubated on ice for 20 min in the dark, followed by two washes with PBS stain buffer. Quantification of cell number counted was determined by CountBright beads (Thermo Fisher). Cells were fixed with 1% formaldehyde in PBS and analyzed on a BD Biosciences FACS Canto instrument within 3 days. FlowJo was used for data analysis.

### 2.4 Antiviral assay

Four hundred and fifty µl of NALF was incubated at room temperature for 30 min with 50 µl of Influenza A/X‐31 (H3N2) (630 EID_50_). After 30 min, 450 µl of the nasal wash plus Influenza virus was applied to confluent Madin‐Darby Canine Kidney (MDCK) cells in 6‐well plates, with each NALF sample in triplicate. The plates were incubated at 37°C in 5% CO_2_ for 60 min with gentle rocking. Control wells contained 450 µl of sterile saline with the same amount of virus, and the positive antiviral control contained 450 µl of sterile saline with 2% ethanol. After 60 min, the nasal lavage and virus solution were removed from wells and plates were washed gently two times with DMEM supplemented with 1% penicillin and streptomycin, followed by a standard influenza plaque assay. Briefly, cells were overlaid with DMEM media containing 1% penicillin and streptomycin, 36% agarose, and 0.2% TPCK‐trypsin. Plates were then incubated at 37°C in 5% CO_2_ for 72 h. After removal of the agarose overlay media, the monolayer was stained with crystal violet and the plaques were counted. The plaque assays for different participants were run on different dates. Therefore, day to day variability was minimized by an assay set‐up in which each participant served as their own control, with each assay including the following wells: saline control, positive control of 2% ethanol diluted in saline, NALF collected at rest, NALF collected after 45 min of exercise, and NALF collected after 180 min of exercise.

### 2.5 Mice and exercise treatment

Adult male BALB/c mice (4 months of age) were randomly divided into the following treatment groups (*n* = 8–10 per group): No exercise (Rest), 45 min of exercise, 90 min of exercise, and 180 min of treadmill running exercise. In an exercise and respiratory viral challenge mouse model, we have previously demonstrated that 45 min of moderate exercise tended to minimize morbidity/mortality from viral challenge, whereas 180 min of exercise resulted in greater morbidity and mortality (Davis et al., 1997), and therefore these exercise durations were selected for their potential clinical relevance. After 3 days of brief acclimation to treadmill running for 10–15 min per day, the mice completed one exercise bout at an average speed of 12–14 meters per minute (moderate‐intensity) for the indicated time. Within 15 min of completing the exercise bout, mice were euthanized by cervical dislocation. Bronchoalveolar lavage (BAL) was performed to collect cells. BAL cells were counted and then used to identify specific subpopulations. This same paradigm and treatment groups (*n* = 4–5 per group) were utilized for subsequent gene expression studies in lung tissue. The study was approved by the Institutional Animal Care and Use Committee and all procedures were carried out in accordance with institutional guidelines for the care and use of animals.

### 2.6 Bronchoalveolar lavage cell populations

The BAL cell populations were defined as follows: alveolar macrophages (high autofluorescence, CD11c^+^, CD11b^−/low^, Ly6G^−^), plasmacytoid dendritic cells (pDCs; CD11c^+/int^, mPDCA^+^, low autofluorescence), and neutrophils (CD11c^−^, CD11b^+^, Ly6G^+^). The following antibodies were used to determine marker positivity: PE‐conjugated anti‐mouse Ly6G (0.25 µg, clone 1A8, Biolegend), APC‐conjugated anti‐mouse mPDCA (1 µg, clone JF05‐1C2.4, Miltenyi Biotec), PE‐Cy‐7‐conjugated anti‐mouse‐CD11c (0.06 µg clone HL3, BD Biosciences), and Alexa Fluor 700‐conjugated anti‐mouse CD11b (0.25 µg, Clone M1/70, Thermo Fisher).

### 2.7 Gene expression

Lung lobes were collected for RNA isolation. RNA was isolated from the lungs using TRIzol reagent (Invitrogen) and further purified using the RNeasy Micro Kit (Qiagen). The RT^2^ first strand kit was used for cDNA synthesis and genomic DNA elimination (Qiagen). Quantitative PCR was performed using the Stress and Toxicity Pathway Finder RT² Profiler™ PCR Array (Qiagen) on a BioRad MyiQ instrument as per the manufacturer's instructions. This specific PCR was selected as the panel of genes includes cellular stress markers to determine whether different exercise durations influence cellular stress and apoptotic pathways or induce a potential inflammatory response. In addition, our focus included heat shock proteins as changes in heat shock protein have been documented with exercise, but not well‐studied within the respiratory tract. The results of gene expression studies are shown in heatmaps prepared with GraphPad Prism software v8.

### 2.8 Statistics

Paired *t*‐tests were used to compare the effects of rest condition versus 45 min of exercise or 180 min of exercise as well as pre‐ versus post‐exercise in human subjects. In murine experiments, differences between groups were determined using one‐way ANOVA. If ANOVA analyses indicated a significant difference, Tukey's post hoc tests were conducted to evaluate differences between groups. The differences and statistical assessment in gene expression were computed using the comparative threshold (ΔΔC_T_) method and pair‐wise comparisons with Super Array software. Data are displayed as mean ± SEMs. A value of *p* < 0.05 was considered statistically significant, and *p* values between 0.05 and 0.10 were reported as trends with respective *p* values presented. All statistical analyses were conducted through SPSS (IBM Corp.). Figures were generated through GraphPad Prism (GraphPad Software).

## 3 RESULTS

### 3.1 Human research participants and exercise

In total, 18 subjects participated in the research study (8 females, 10 males) with ages ranging from 18 to 33. Twelve participants completed the 45‐min exercise session (six females, six males) while eight (four females, four males) completed the 180‐min exercise session. No participants delayed a schedule visit due to illness symptoms. Participant information and the response to exercise are shown in Table 1. As expected, fatigue score significantly increased after the 45‐ and 180‐min exercise conditions relative to pre‐exercise fatigue score.

**TABLE 1:** Reported fatigue increased post‐exercise relative to the pre‐exercise measurement

Demographics and responses to exercise	45 min	180 min
Age (years)	22.6 ± 3.5	22.2 ± 2.4
Mean heart rate (bpm)	146.7 ± 6.2	138.7 ± 3.6
Pre‐exercise fatigue rating (cm)	3.0 ± 3.3	2.0 ± 1.4
Post‐exercise fatigue rating (cm)	10.1 ± 3.3*	16.6 ± 2.6*
Mean RPE rating	12.4 ± 1.3	13.0 ± 0.8

Note

Participant fatigue rating by the visual analog fatigue scale (measured in cm) was assessed prior and following respective exercise conditions. Data are represented as mean ± standard deviation.

* *p* < 0.05 by paired *t*‐test.

### 3.2 Prolonged exercise alters NALF total cellular and leukocyte counts, which significantly correlates with reported fatigue

**FIGURE 1:**
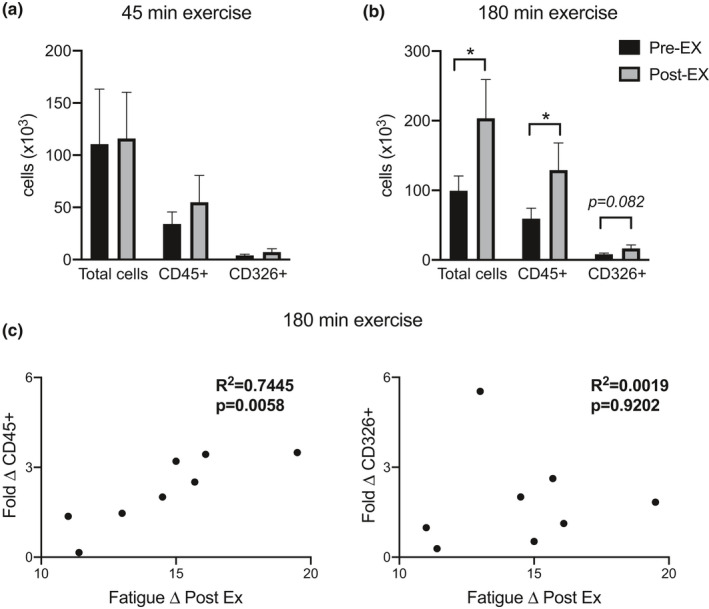
Prolonged exercise alters nasal lavage fluid (NALF) total cellular and leukocyte counts, which significantly correlates with reported fatigue. NALF was collected before and after 45 and 180 min of exercise from human subjects. NALF was assessed for total, leukocyte (CD45^+^), and epithelial (CD326^+^) cell counts after (a) 45 min of exercise (*n* = 18) or (b) 180 min of exercise (*n* = 8). (c) Cell count fold change was correlated with post‐exercise fatigue by visual analog scale. *p < 0.05, significance by paired *t*‐tests or Pearson's correlation (two‐tailed)

The total number of cells recovered was significantly increased by exercise condition depending on the duration. After 45 min of exercise, there was no change in total number of cells recovered in the NALF, percentage of leukocytes (CD45^+^), or epithelial cells (CD326^+^; Figure 1a). However, after 180 min of exercise, there was a statistically significant increase in the total number of cells, the total number of leukocytes, and a trend for an increase in epithelial cells (Figure 1b). Cell populations and flow cytometry gating are shown in Figure S1. The change in leukocyte numbers may reflect alteration of cell trafficking or adherence, whereas the epithelial cell trend may reflect changes in cell adherence or shedding.

Given the interest in identifying immune markers that may be associated with exertion, a correlation between the change in fatigue in response to 180 min of exercise and change in NALF CD45^+^ and CD326^+^ cells was assessed. After 45 min of exercise, there were no associations between any of the cell populations and fatigue (data not shown). In contrast, after 180 min of exercise the increase in NALF CD45^+^ cells was significantly correlated with the increase in fatigue (Figure 1c). The CD326^+^ cell response to 180 min of exercise was not significantly correlated with fatigue (Figure 1c).

### 3.3 Acellular NALF has antiviral activity at rest and post‐exercise

**FIGURE 2:**
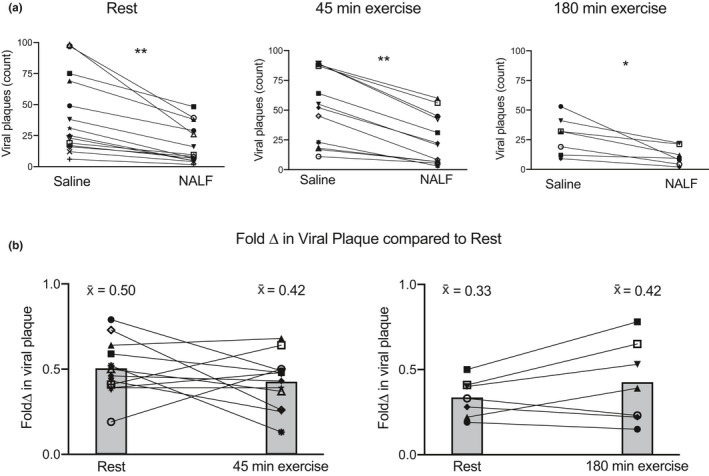
Nasal lavage fluid (NALF) has antiviral activity compared to saline, independent of exercise, while exercise duration potentially alters antiviral activity. Normal saline or NALF from human subjects at rest or during exercise was assessed for antiviral activity. (a) Saline or NALF from exercised subjects was incubated with Influenza A virus followed by addition to MDCK cells for plaque assay. (b) Fold change in viral plaque formation was assessed between rest and exercise duration conditions. **p < 0.01, *p < 0.05 by paired *t*‐test; x̄ = sample mean

The fluid portion of NALF was used to assess the antiviral activity by pre‐incubating Influenza A virus with either normal saline or NALF, followed by addition to MDCK cells for plaque assay. NALF alone contributes significant antiviral activity, as evidenced by the reduction in number of viral plaques (representing the number of infectious viral particles) with the addition of NALF from resting participants (Figure 2a). This antiviral activity was also present in NALF collected after 45 min of exercise (Figure 2a) and after 180 min of exercise (Figure 2a). In order to directly compare the change in antiviral activity due to exercise in each participant, the fold change in plaque reduction relative to saline control was determined for each participant, which reduced the confounding factor of assay variability. In this fold change calculation, a lower number represents a greater decline in virus and indicates increased antiviral activity. After 45 min of exercise, a pattern of reduced plaque number (greater antiviral activity of NALF), relative to the rest condition was apparent in 8 of 12 participants (Figure 2b). However, the mean fold reduction (0.50 at rest to 0.42 post‐exercise) was not statistically significantly different, and in a subset of participants (*n* = 3, 25%) the opposite trend was observed (i.e., greater viral growth with NALF post‐exercise). The only difference between participants who exhibited a reduction in viral load (*n* = 8) compared to those who showed no change or an increase (*n* = 4), was the post‐exercise fatigue score [score = 11.7 ± 2.8 in participants with reduced viral load, and score = 7.1 ± 1.9 in participants with increase or no change in viral load (mean ± SD)]. In response to 180 min of exercise, the pattern of fold change in plaque reduction appeared reversed, with a tendency toward increased viral plaques post‐exercise relative to the rest condition (Figure 2b). Yet, this apparent mean decline in antiviral activity after 180 min of exercise was not significantly different than the resting condition (Figure 2a).

### 3.4 BAL cells and lung gene expression in response to 45, 90, or 180 min of exercise

**FIGURE 3:**
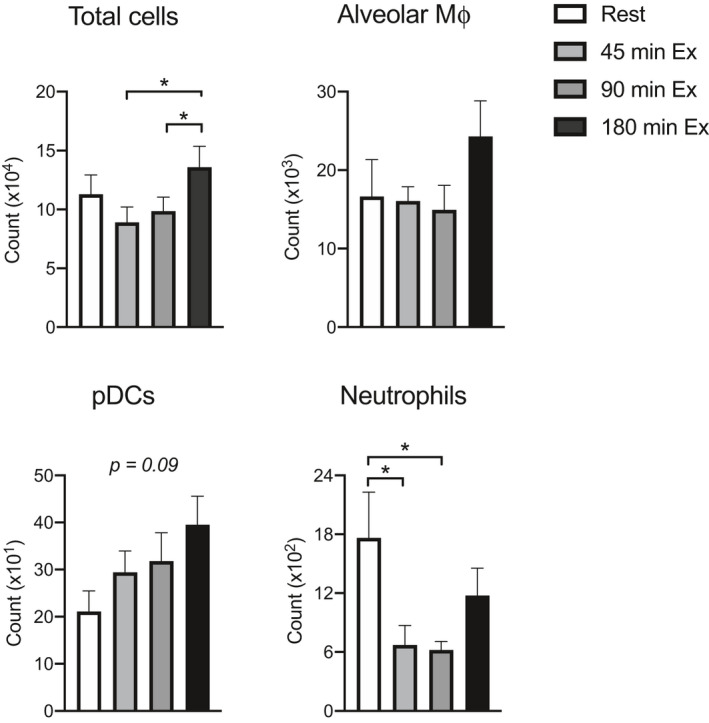
Exercise duration alters bronchoalveolar lavage (BAL) fluid total and specific cellular counts. Mice (*n* = 8–10) completed an acute treadmill running exercise bout of varying durations followed by bronchoalveolar lavage. Cells were filtered and stained for total and specific population counts by flow cytometry. *p < 0.05 with bars indicating one‐way ANOVA with post hoc Tukey's test between respective groups; italicized *p* value indicates main effect trend. pDC, plasmacytoid dendritic cells; mΦ, macrophage

As the assessment of lower respiratory tract responses requires invasive procedures in humans, a mouse model was utilized to examine changes in bronchoalveolar lavage cell populations and changes in gene expression within whole lung lobes. In BAL fluid, total cell count increased in 180 min of exercise as compared to 45 and 90 min, but not rest (Figure 3). In examining specific cellular subtypes, pDCs and alveolar macrophage populations were unchanged by exercise paradigms in mice, although trended to increase at 180 min of exercise (Figure 3). Interestingly, there was a marked reduction in neutrophils from rest to 45‐ and 90‐min exercise paradigms, with neutrophil counts returning to comparable levels to resting after 180 min of exercise (Figure 3).

**FIGURE 4:**
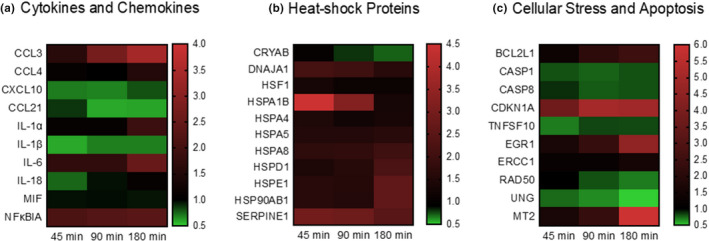
Exercise duration exerts complex effects on lung gene expression of inflammatory, heat‐shock related, and cellular stress pathways. Mice (*n* = 8–10) completed an acute treadmill running exercise bout of varying durations. Lung lobes were then harvested followed by RNA extraction and cDNA conversion followed by gene expression analysis by qPCR. Heatmap corresponds to gene expression fold change relative to resting condition at 1.0 (baseline control). Fold change in gene expression is shown to the right of each figure

With respect to gene expression in whole lung lobes, microarrays were used to assess gene categories relating to inflammatory cytokines and chemokines, heat‐shock related proteins, and apoptosis/cellular stress pathways. The fold change in gene expression was calculated as relative to no exercise baseline expression, such that a value of 1.0 indicates control levels. The genes displayed in Figure 4 showed statistically significant differences in expression (*p* < 0.05) in one or more pair‐wise comparisons relative to no exercise, except for the following genes, CCL4, CXCL10, IL‐1β, MIF, and IL‐18, as these genes were included in the table to illustrate minimal effects of exercise on several inflammation‐related genes. The time point(s) that were significantly different from no exercise varied by gene. The heatmap of inflammation‐related cytokines or chemokines demonstrates inflammatory cytokines or chemokines were generally downregulated or did not change in response to exercise, with a trend toward a decrease in IL‐1β and IL‐18 (*p* value between 0.05 and 0.10) (Figure 4a). However, a modest upregulation of IL‐6 and IL‐1α was measured at 180 min of exercise, and the expression of chemokine CCL3 (MIP‐1α) was significantly upregulated at all three times points with a dose‐dependent relationship to exercise duration. NFκBIA remained modestly but consistently elevated at all exercise time points, potentially reflecting an anti‐inflammatory effect by keeping NFκB bound in the IKK complex, limiting NFκB activation.

Furthermore, we demonstrate nearly all heat shock‐related proteins were increased by exercise (Figure 4b). The pattern of expression by exercise duration was variable—for example, some genes had greater expression at 45 min of exercise with a gradual decline over time (HSPA1B, SERPINE1), while other genes had a continued increase as exercise duration increased. The expression of pro‐apoptotic genes was decreased with increasing exercise duration (caspases, TNFSF10), whereas genes with anti‐apoptotic activity increased (BCL2L1, CDKN1A) (Figure 4c). Metallothionein (Mt2) had the greatest increase in expression of any gene measured in the microarray, with 6.2‐fold increase after 180 min of exercise. Genes related to DNA damage or repair (EGR1, ERCC1, RAD50, and UNG) showed a mixed response to increasing exercise duration with EGR1 and EGCC1 modestly upregulated but a downregulation in RAD50 and UNG. Notably, early growth response 1 (EGR1) expression was modestly increased in lung; it is known to be increased in muscle tissue after exercise (Chen et al., 2002).

## 4 DISCUSSION

In the present study, NALF collected from participants after 180 min of exercise had increased leukocytes and total cells and correlated strongly with post‐exercise fatigue ratings. In both exercise and resting conditions, the acellular portion of NALF had antiviral activity against Influenza A. In moderately exercised mice, neutrophils were found to be decreased in BAL. In the whole lung, increases in gene expression of cellular stress and inflammatory proteins were observed that varied by exercise duration.

To our knowledge, these are the first results to demonstrate prolonged exercise increases the number of leukocytes in NALF. The change in cell number takes place after 180 min of exercise, but not after 45 min of moderate exercise. Others have demonstrated an increase specifically in the NALF neutrophil population following a long‐distance race (Müns et al., 1995). In studies of induced sputum after longer endurance events, an increase in both leukocytes and bronchial epithelial cells has been reported (Bonsignore et al., 2001, 2003; Chimenti et al., 2010; Seys et al., 2015). Therefore, our findings with respect to the effects of long endurance exercise NALF cell populations are similar to the findings on induced sputum.

Leukocytes, including neutrophils, are likely to be recruited to the nasal mucosa or become less adherent during exercise. In contrast to leukocytes, mucosal epithelial cells are a localized cell population that are not recruited and may be shed during exercise. A previous study showed that ciliated epithelial cells from nasal biopsies taken post‐marathon had reduced ciliary beat frequency, along with a greater number of dead ciliated cells or cells with impaired, immotile cilia (Müns et al., 1995). Evidence of bronchial epithelial cell damage has been reported among athletes in several studies (Bougault et al., 2009), but there is less information with respect to the effects of acute exercise. In two studies, the induced sputum obtained either after long‐duration exercise or short intense exercise had an increased number of bronchial epithelial cells (Chimenti et al., 2010; Morici et al., 2004). Several other investigators have assessed lower airway epithelial damage using serum or urine concentration of club cell secretory protein (CC‐16) as a biomarker. This protein is secreted by non‐ciliated cells of the bronchioles, and although it has been used as an indicator of epithelial stress during exercise, there are some concerns as to whether this accurately reflects epithelial stress (Lakind et al., 2007). An increase in CC‐16 has been reported after brief, intense exercise, or prolonged exercise, but this is not a consistent finding (Bolger et al., 2011; Chimenti et al., 2010; Seys et al., 2015; Tufvesson et al., 2013). Taken together, our findings and those of Müns et al. (1995) with respect to the upper airways, and other findings based on induced sputum, indicate epithelial cell damage or shedding may occur with prolonged exercise. Epithelial cells form a protective barrier and exhibit immune defense functions that include the initiation of innate immunity (Günther & Seyfert, 2018; Hariri & Cohen, 2016; Whitsett & Alenghat, 2015). If a loss of nasal mucosal epithelial cells compromises the protective barrier, or the defense functions of epithelial cells are impaired, susceptibility to respiratory infection may be increased following prolonged exercise. This possibility aligns with findings from studies in humans in which increased rates of respiratory infection were noted after a single session of prolonged exercise (Nieman et al., 1990; Peters & Bateman, 1983; Peters et al., 1993) and animal studies showing greater severity of infection when pathogen challenge took place shortly after fatiguing exercise (Davis et al., 1997; Murphy et al., 2008). However, it is important to consider that other host defense mechanisms may serve to counteract an exercise‐induced loss of functional epithelial cells, and these mechanisms may include the antimicrobial peptides present in NALF (Hariri & Cohen, 2016), changes in the mucociliary fluid or its movement, ciliary beat frequency, or a recruitment of immune cells to the nasal mucosa.

Our findings add new information to the existing body of literature by demonstrating that acellular nasal lavage fluid obtained from participants at rest or after moderate or prolonged exercise has direct antiviral activity against Influenza A virus. However, our findings did not clearly delineate whether the antiviral activity of post‐exercise NALF differs from the resting condition. The overall pattern after 45 min of exercise suggested enhanced antiviral defense, while the overall trend after 180 min of exercise indicated reduced antiviral defense, but there was a great degree of interindividual variability in the response to exercise (Figure 2). Given that NALF and virus were incubated on MDCK cells for only an hour prior to washing, NALF may act with direct antiviral activity against influenza, may interfere with virus attachment, or induce an antiviral state. In view of our findings and the results from other studies that demonstrated exercise‐associated respiratory epithelial cell damage or loss, the antiviral activity of NALF may protect against infection. We did not measure anti‐influenza IgA in NALF however, it is not likely that human antibodies directed against the Influenza A/X31 strain would be present. And although our study did not evaluate the contribution of specific AMPs or surfactant proteins (surfactant proteins A and D) in NALF, several AMPs have known antiviral activity against influenza. For example, cathelicidin LL37, α‐defensins, and β‐defensins found in NALF interfere with or inhibit influenza replication (Kalenik et al., 2018; Salvatore et al., 2007; Tripathi et al., 2013). Epithelial cells can secrete LL37 or β‐defensins, and leukocytes are the source of cathelicidins and α‐ or β‐defensins. Therefore, if epithelial cells are either shed or become dysfunctional after prolonged exercise, leukocytes can be a source of AMPs contributing to host defense. Few studies have evaluated the effects of acute exercise on AMPs in the context of mucosal immunity, although an increase in salivary lactoferrin or lysozyme after shorter duration intense exercise has been reported (Allgrove et al., 2014; Ligtenberg et al., 2015; West et al., 2010). Lactoferrin may inhibit influenza virus (Pietrantoni et al., 2010), although we are not aware of anti‐influenza actions of lysozyme. Given that other studies assessed saliva rather than NALF, and utilized shorter duration exercise, it is difficult to directly compare with our results. However additional studies are warranted to determine which AMPs in NALF may exhibit anti‐influenza activity, and the extent to which their function may be altered during exercise.

In examining the cellular fraction of NALF, our findings suggested leukocyte recruitment to the nasal mucosa occurs after 180 min of exercise. Others have observed increased neutrophils in NALF after completing a marathon, along with increased chemotactic activity for neutrophils (Müns et al., 1995). A novel finding from our experiments was the significant correlation between reported fatigue level and the increase in NALF CD45^+^ leukocytes in response to 3 h of exercise. The mechanism responsible for the association between fatigue level and extent of NALF leukocyte increase was not identified in this study, but several possibilities exist that remain speculative at this point. Prolonged exercise at a moderate intensity is accompanied by glucocorticoid release. Glucocorticoids have metabolic effects, yet glucocorticoids may also downregulate L‐selectin, or enhance L‐selectin shedding, contributing to increased circulating neutrophils (Weber et al., 2004). A correlation between the number of neutrophils in induced sputum and plasma cortisol post‐marathon has also been reported, providing support for this possibility (Bonsignore et al., 2001). Dopamine and serotonin contribute to central fatigue with long endurance exercise (Cordeiro et al., 2017). Although the relationship between peripheral and central levels of these neurotransmitters during exercise is complex, leukocytes express receptors for these neurotransmitters as well as synthesize their own (Elkhatib & Case, 2019). Important leukocyte functions such as rolling may be altered by serotonin or dopamine (Cordeiro et al., 2017; Herr et al., 2014; Thomas Broome et al., 2020). Also, because there is increased shedding of epithelial cells, the loss of such cells may trigger chemokine or other chemotactic factor release that promotes neutrophil infiltration. Further research is needed to define the mechanisms that may underlie the relationship between reported fatigue after prolonged exercise and leukocyte number in NALF. We also note an interesting observation between NALF antiviral activity after 45 min of exercise and reported fatigue. The antiviral activity in NALF from most participants was greater after exercise, but in four participants no change or a decrease in antiviral activity of NALF was found. The average fatigue score post‐exercise in these four individuals was significantly lower than the other participants, which raises the possibility that some threshold in the perception of exercise intensity may be necessary for the activation of antiviral responses. However, this possibility remains speculative and requires further study.

Few studies have examined the immune response to acute exercise in the upper airways. Instead, there has been a greater focus on the lower airways and EIB. Elite athletes may experience airway epithelial injury during exercise under certain conditions that may ultimately contribute to EIB (Anderson & Kippelen, 2008). Although the effect of exercise on airway response has been evaluated in human participants, it has often been studied in the context of EIB or elite athletes and limited to induced sputum. Other methods to evaluate lower airway response such as BAL collection or biopsies are invasive procedures. Therefore, given the challenges in assessing lower airway response to exercise, we used a mouse model to evaluate the effects of exercise. With respect to BAL cells, the number of cells recovered varied by exercise time point, an initial trend toward a decline and perhaps trafficking away from the airways, but an increase by 180 min of exercise. The relatively rapid change in neutrophil number may reflect the trafficking of these cells to other locations. These findings in the mouse model parallel the results from human trials of induced sputum in which leukocyte populations’ shifts also occur post‐exercise (Bonsignore et al., 2001, 2003; Seys et al., 2015), along with a decrease in the number of neutrophils expressing L‐selectin. The change in L‐selectin expression would be expected to influence cell trafficking as could also enhanced neutrophil expression of beta2 integrins and/or endothelial and epithelial cell expression of ICAM‐1. To our knowledge, the effect of exercise on pDC number in the respiratory tract has not yet been determined in humans or animal models. pDCs secrete vast amounts of interferon‐alpha in response to endosomal TLR activation, thereby providing early antiviral defense (Swiecki & Colonna, 2015). Our results show a potential trend (*p* = 0.09) toward increased pDCs as exercise duration increases, potentially reflecting pDC recruitment to the airways to serve an antiviral role if epithelial barriers are compromised. Based on our findings and the existing literature, it appears that acute exercise causes a shift in leukocyte populations of the upper and lower airways, but the extent to which this shift alters susceptibility to infection requires further study.

The gene expression studies suggest a cellular stress response in the lungs as heat shock proteins’ expression was upregulated by exercise. Heat shock transcription factor (HSF1) was modestly induced by exercise, and its induction can lead to the expression of a large class of heat shock proteins. An increase in HSPs in response to acute exercise has been widely recognized (Henstridge et al., 2016), although, to our knowledge, these are the first results to demonstrate increased expression in the lung. The cell types expressing HSP were not determined. Still, because there may be loss of epithelial cells and thus epithelial shedding, the stress in epithelial cells may contribute to increased HSP production. HSPs have a complex role in immune regulation and may serve as danger‐associated molecular patterns (DAMPs) to induce toll‐like receptor (TLR) activation resulting in an inflammatory response (Asea et al., 2002). Conversely, HSPs may limit the inflammatory signaling cascade in macrophages and influence the formation of Iκκ complex, thus preventing the translocation of NFκB, thereby limiting inflammation in respiratory epithelial cells (Chen et al., 2006; Yoo et al., 2000). HSF1 induction may limit IL‐1β transcription (Xie et al., 2002), consistent with the downregulation of IL‐1β that we observed (Figure 4a). Our gene expression results do not support a potent inflammatory response in the lungs with long‐term exercise. Cross talk between HSPs and downstream inflammatory signaling pathways may take place to limit inflammation. Of the chemokines that influence cell trafficking, most were downregulated or had no change, but CCL3 continued to increase as exercise duration increased. CCL3 may bind to receptors CCR1, CCR4, and CCR5, and the expression of these chemokine receptors varies widely across immune cell subsets. Therefore, many different cell types may respond to CCL3, including monocytes, T‐helper 2 cells, regulatory T cells, neutrophils, natural killer cells, T‐helper 1 cells, and pDCs. Although changes in cell trafficking occurred, there was no substantial evidence of an inflammatory response; as a classic inflammatory cytokine, IL‐1β was not upregulated. NFKBIA, which inhibits the activity of NFκB‐Rel complexes, was significantly upregulated by 90 or 180 min of exercise. IL‐6 increased at the 180‐min exercise time point, but IL‐6 may serve a metabolic role during exercise, similar to the response in other tissues (Hennigar et al., 2017), rather than inflammatory activity. It is also possible that an inflammatory response could occur in the exercise recovery period, although we did not assess later time points. In the context of potential infection, 3 h of increased ventilation could increase exposure to pathogens, and therefore immune activation may be beneficial. However, an ongoing inflammatory state in the airways could have detrimental effects, and one could speculate that a quick return to an anti‐inflammatory environment post‐exercise might be optimal. In studies of athletes that exercise regularly or animal models of exercise training, there are reports of increased leukocyte populations in the lung, including total leukocytes, neutrophils, lymphocytes, eosinophils, mast cell, and macrophages (Bonsignore et al., 2001, 2003; Chimenti et al., 2007; Denguezli et al., 2008; Helenius et al., 1998; Sue‐Chu et al., 1999). However, these changes in cell populations are not associated with changes in pulmonary function or inflammatory activation (Bonsignore et al., 2003; Chimenti et al., 2007; Denguezli et al., 2008). In fact, in the context of allergic airway response, inflammatory challenge, or resistance to respiratory infection, regular exercise training reduces the inflammatory response (Reis Gonçalves et al., 2012; Silva et al., 2010; Sim et al., 2009; Vieira et al., 2011). In general, our findings also suggest a decrease in apoptotic signals, with downregulation of CASP8, TNFSF10, and CASP1 (pyroptosis‐inducing), but an upregulation of apoptosis inhibitor‐related genes BCL2L1 and CDKN1A. The possibility that cellular stress due to prolonged exercise might induce apoptosis is not supported by our results. We also evaluated several genes important in DNA damage or repair process, yet mixed results were found, and further research is needed to clarify the impact of long‐duration exercise on DNA damage or repair pathways. It is of interest to note others have reported an increase in ERCC1 after exercise and have hypothesized that this may be one mechanism contributing to extended life span in individuals who exercise (Ji et al., 2019).

There are several limitations of this investigation that are important to consider. In the human exercise paradigm, eight subjects elected to complete the prolonged (180 min) exercise bout. The inclusion/exclusion criteria for participation in either protocol was identical, but it is possible that subjects who were more aerobically fit selected to complete both conditions, or the 180‐min condition. Based on heart rate at a given workload, differences in fitness of participants in the 45‐min condition or the 180‐min condition were not apparent, but V0_2_max was not assessed, and it remains possible that fitness differences existed.

Overall, several new findings from this study expand our knowledge concerning exercise and mucosal immunity. We provide new evidence that demonstrates NALF collected after moderate or long‐duration exercise contains antiviral activity against Influenza A virus, and the shift in leukocytes to the nasal mucosa is significantly correlated with fatigue after 180 min of exercise. New findings from the studies with mice demonstrate a consistent pattern of upregulation in HSP gene expression in the lungs in response to exercise, suggestive of cellular stress. Other results presented are generally consistent with the existing albeit limited literature, including an exercise‐induced shift in leukocyte populations in the airways. Still, to our knowledge, the findings with respect to the pDC population are new and require further study. Moreover, our data suggest that long‐duration exercise impacts respiratory mucosal epithelial cells in parallel with other studies. These findings do not completely resolve the questions that have been raised regarding the "open window" theory of vulnerability to infection after exercise. However, the results provide some evidence to suggest cellular stress and epithelial damage may occur with prolonged exercise. Nevertheless, protective mechanisms such as antiviral AMPs in mucosal fluid or the recruitment of leukocytes to the upper airways may counter the stress or damage and help to protect against pathogens.

## ETHICS

This study was carried out in accordance with the recommendations of the Iowa State University Institutional Animal Care and Use Committee and Institutional Review Board. All protocols were approved by the Iowa State University Institutional Animal Care and Use Committee and Institutional Review Board.

## CONFLICT OF INTEREST

The authors have declared that no conflict of interest exists.

## AUTHOR CONTRIBUTIONS

MRA, DEV, MLK, and SN designed the research methods and experimental approach. SKE, JA, MJ, LS, AB, SN, and MLK conducted the experiments and analyzed the data. SKE and MLK wrote the manuscript. All authors reviewed, edited, and approved the manuscript. MLK provided primary experimental oversight.

## Supporting information



Fig S1

## Data Availability

The data that support the findings of this study are available from the corresponding author upon reasonable request.
