# Supplementary material for: Exercise duration modulates upper and lower respiratory fluid cellularity, antiviral activity, and lung gene expression
Source: Physiol Rep. 2021 Oct 21;9(20):e15075. doi: 10.14814/phy2.15075 (PMC8531599; doi:10.14814/phy2.15075)
Supplement: Supplementary file 1 — Fig S1 [file PHY2-9-e15075-s001.docx]

A. Cells collected in the rest condition

**Supplement Fig 1**. Flow cytometry gating. Cells in NALF from the same individual are shown in the Rest condition (A) and after 180 minutes of exercise (B). CountBrite beads are indicated in red. Cell gates and CD45^+^ or CD326^+^ cell populations are shown.

B. Cells collected after 3 hours of exercise
